# Sarcoplasmic reticulum is an intermediary of mitochondrial and myofibrillar growth at the intercalated disc

**DOI:** 10.1007/s10974-016-9444-6

**Published:** 2016-06-21

**Authors:** Pauline M. Bennett, Elisabeth Ehler, Amanda J. Wilson

**Affiliations:** 1 Randall Division of Cell and Molecular Biophysics, King’s College London, New Hunt’s House, Guy’s Campus, London, SE1 1UL UK; 2 Department of Life Sciences, Sir Ernst Chain Building, Imperial College London, South Kensington Campus, London, SW7 2AZ UK

**Keywords:** Dilated cardiomyopathy, Heart, Mitochondria, Intercalated disc, T tubules, Electron tomography

## Abstract

In cardiomyocytes columns of intermyofibrillar mitochondria run up to the intercalated disc (ID); half are collinear with those in the neighbouring cell, suggesting coordinated addition of sarcomeres and mitochondria both within and between cells during cardiomyocyte growth. Recent evidence for an association between sarcoplasmic reticulum (SR) and mitochondria indicates that the SR may be an intermediary in this coordinated behaviour. For this reason we have investigated the arrangement of SR and t tubules with respect to mitochondria and myofibrils, particularly at the ID. In the body of the cardiomyocyte the mitochondrial columns are frequently intersected by transverse tubules. In addition, we find that a majority of axial tubules are sandwiched between mitochondria and myofibril. No tubules are found at the ID. SR coats mitochondrial columns and fibrils throughout their length and reaches towards the peaks of the ID membrane where it attaches in the form of junctional (j)SR. These peripheral ID couplings are often situated between mitochondria and ID membrane, suggesting an SR connection between the two. In dilated cardiomyopathy (DCM) the mitochondria are somewhat disordered and clumped. In a mouse model for DCM, the muscle LIM protein KO, we find that there is a lack of mitochondria near the ID, suggesting the uncoupling of the myofibril/mitochondria organisation during growth. SR still coats the fibrils and reaches the ID folds in a jSR coupling. Unlike in control tissue, however, loops and long fingers of ID membrane penetrate into the proximal sarcomere suggesting a possible intermediary state in cardiomyocyte growth.

## Introduction

**Fig. 1:**
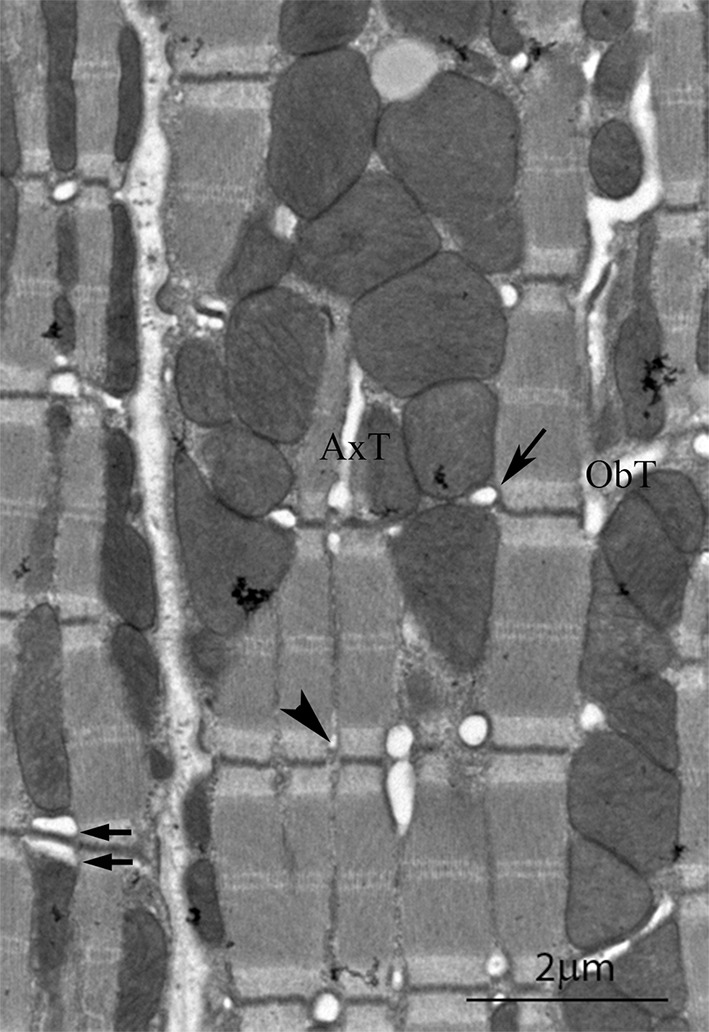
Electron micrograph of a longitudinal section of mouse *left* ventricle showing details of the t tubule system. In the cell on the *left* the continuity of mitochondrial columns is seen. The cell on the *right* is sectioned slightly obliquely. (*arrow*) Transverse tubule of larger diameter at the mitochondria/myofibril boundaries; (*arrowhead*) smaller diameter tubule at the myofibril/myofibril boundaries. (*arrow pair*) double transverse tubule; ObT, oblique tubule; AxT, axial tubule

The cardiomyocyte mainly comprises parallel arrays of myofibrils and mitochondria. Periodically intruding into this array are the t-tubules and the associated sarcoplasmic reticulum (SR) (Fig. 1) (Fawcett and McNutt 1969; Ayettey and Navaratnam 1978). Recent observations have revealed an important relationship between mitochondria and endoplasmic reticulum (SR in muscle), the implications of which have not been fully examined in muscle structure studies.

In cells, in general, a close association between mitochondria and endoplasmic reticulum (ER) has been shown to be necessary to deliver to mitochondria, locally, a high concentration of calcium for efficient production of ATP and control of oxidative processes (Pizzo et al. 2012). Physical links some 10–25 nm long are seen between mitochondria and ER in rat liver (Csordas et al. 2006). Further, ER is implicated in fission of mitochondria, a process important for cell health (da Silva et al. 2014). Mitochondria divide at positions where ER contacts and wraps around them in a process involving dynamin-like protein 1 and actin (Friedman et al. 2011; Lackner et al. 2013; Marchi et al. 2014).

In muscle, the role of SR, the ER equivalent, in excitation–contraction coupling is well known. Part of it, junctional SR (jSR), is coupled to t-tubules (invaginations of the plasma membrane) and plasma membrane, forming couplons or calcium release units (CRUs) (Franzini-Armstrong et al. 1999). In the heart, the CRU involves a close proximity between ryanodine receptors (RyR2) on the SR and CaV_1.2_, the L type Ca channel on the t tubule The rest of the SR, free SR, forms an anastomosing stocking around the myofibrils (see for example Sommer and Waugh 1976; Pinali et al. 2013). This SERCA rich organelle pumps calcium out of the cytoplasm and keeps the general level of calcium at a necessary low for both cell health and myofibril relaxation. Evidence for ER/mitochondrial calcium crosstalk strongly suggests an intimate functional relationship in muscle between mitochondria, SR and t-tubule. Franzini-Armstrong and her colleagues have shown that in the rat ventricle some 90 % of CRUs are close to mitochondria (Ramesh et al. 1998; Sharma et al. 2000). Further, physical links about 10 nm long between SR and mitochondria have been revealed in skeletal muscle near to triads and in cardiac muscle between mitochondria and both free and jSR (Boncompagni et al. 2009; Hayashi et al. 2009).

The high energy demands of the heart mean that a large volume, some ~30–35 % of the cardiomyocyte, comprises mitochondria, most of which are in intermyofibrillar columns. This intimate relationship allows for a ready supply of ATP for muscle contraction. In ventricular cells these columns are frequently intersected by transverse tubules at the Z-disc level. Electron microscopy has shown that the t tubule system in the heart is complex, comprising both transverse, oblique and axial components (Fawcett and McNutt 1969; Forssmann and Girardier 1970; Sperelakis and Rubio 1971; Forbes et al. 1984; Tomita and Ferrans 1987; Forbes and van Neil 1988). A transverse system of relatively large tubules spreads from the plasma membrane into the cardiomyocyte at the Z-disc level separated axially by ~2 μm, the sarcomere repeat (Kostin et al. 1998). These transverse elements are often branched and also connected by longitudinal or oblique tubules. The intricacies of the three dimensional tubule array within a cardiomyocyte have been revealed using high resolution immunofluorescence (Soeller and Cannell 1999; Wagner et al. 2012) and serial sectioning combined with scanning electron microscopy (Pinali et al. 2013). However, these detailed observations do not reveal the general relationship of t tubules with mitochondria. We have reassessed our images of ventricular myocytes in the mouse to understand this organisation.

Little is reported of the role of mitochondria at the intercalated disc (ID). The IDs at the ends of cardiomyocytes are dynamic regions rich in folded membrane which interdigitate with the membrane of neighbouring cells. The margin between the contractile and ID regions of the cell, the transitional junction (TrJ), is very precise and acts as a proto Z-disc for the proximal sarcomere (Bennett et al. 2006; Bennett 2012). It has been suggested that cardiomyocytes can control their length to some extent by increasing or decreasing the size of the ID folds (Yoshida et al. 2010; Wilson et al. 2014). For greater growth there is evidence for the interpolation of new sarcomeres within the ID folds when they become sufficiently large. The organisation of fibrils at the ID is therefore relatively well understood but the relationship of other organelles, in particular, mitochondria, t tubules and SR to the ID has not been described in detail. We have sought to understand this organisation using electron microscopy and electron tomography. We have found that the mitochondrial columns continue to the edge of the ID, and we suggest that elongation of mitochondrial columns and myofibrils, during cardiomyocyte growth, is coordinated both within and between cells. Further, the close relationship of SR with both fibrils and mitochondria extends to its connection with the ID membrane fold.

There are a number of reports of reorganisation and loss of function of both mitochondria and t tubules in heart disease. Disruption of tubule organisation in heart disease has been reported in heart failure, dilated cardiomyopathy (DCM) and hypertrophic cardiomyopathy (HCM) (Ibrahim et al. 2011; Wagner et al. 2012; Ferrantini et al. 2013; Pinali et al. 2013). This disruption can take the form of a loss of transverse tubules and their regular openings at the sarcolemma, and an increase in the number of axial and oblique tubules. Alternatively, t-tubules can become distended as has been reported in the desmin ko mouse with an ARVC phenotype (Thornell et al. 1997) and the Bin1 KO mouse leading to ventricular arrythmia (Hong et al. 2014).

For mitochondria, disorganisation in heart disease is revealed both by clumping and loss and by a reduction in size that may be symptomatic of more fission than fusion (Ong and Hausenloy 2010; Dorn 2013). Knock out of mitofusin 1 or 2 leads to mild hypertrophy and changes in the size of mitochondria (Papanicolaou et al. 2011, 2012). An SR link to mitochondria is supported in skeletal muscle, where loss of longitudinal SR in an obscurin KO mouse is accompanied by an anecdotal loss of mitochondria (Lange et al. 2009).

To study changes in the relationship of mitochondria, t tubules and SR in DCM, we used a mouse model for a non-lethal form, the muscle LIM protein (MLP) KO mouse (Arber et al. 1997). These mice develop dilated hearts with the expression of hypertrophic marker proteins a few weeks after birth, which appear to stabilise at maturity. The mice are viable and survive to normal old age. Changes have been reported in the mitochondrial distribution including clumping and/or loss, together with alterations in function (van den Bosch et al. 2005; Wilding et al. 2006). Here, we show that despite the changes there was no loss of mitochondrial volume or obvious change in relation to t-tubules. However, there was a reduction in mitochondria number at the ID in the MLP KO heart (Wilding et al. 2006; Wilson et al. 2014). We have characterised this effect and suggest that this absence indicates a loss of coordination of fibril and mitochondrial column growth and indeed a breakdown in communication with the neighbouring cells. The SR relationship with fibrils at the ID folds remained. However, unlike in control cells some long fingers of ID fold were found in the fibril compartment and met the SR half way. This observation modifies our model for cell growth and sarcomere insertion.

## Methods

### Specimen preparation

Mice were maintained under Home Office regulations, Project Licence 70/06495 in accordance with the Animal Scientific Procedures Act, UK, 1986.

Sections from mouse heart were prepared as previously described (Wilson et al. 2014). The types of specimen used were the mouse model of DCM, the MLP KO, and its control strain, SV129. For some of the tomography, specimens were high-pressure frozen. Hearts taken from MLP KO and control mice were fixed in 4 % PFA in PBS and washed in PBS as standard procedure. 100 μm sections were cut on a Leica Vibratome 1000S and then small pieces were high pressure frozen in a Leica EM PACT. Frozen samples were freeze substituted in 2 % osmium in acetone, washed in acetone and embedded in Araldite (Bennett et al. 2002).

### Electron microscopy and tomography

For routine microscopy of thin sections, a Hitachi H7600 was used.

For tomography 200–400 nm thick sections of standard or freeze substituted blocks were lightly stained in ethanolic UA and 10 nm gold particles used as fiducials. Micrographs were obtained in an FEI Tecnai T20 with a LaB6 filament at 200 keV. Tilted series at 1^o^ degree intervals up to ± 70^o^ in two perpendicular directions were obtained. Tomographic reconstructions were computed using IMOD software (Kremer et al. 1996; Mastronarde 1997). The same software was used to segment the images.

Electron microscopy was carried out in the Centre for Ultrastructural Imaging, King’s College London.

### Measurements

The density of axial tubules was determined from images of transverse sections of cardiomyocytes. Each cell image was printed and the axial tubules in A-band regions were identified and counted. Their position with respect to mitochondria was also noted. The image was overlaid with a grid, and the number of intersections of the grid with A-band regions compared to the total was established. From this number the area of A band was determined from the total area of the cell determined in Photoshop.

For analysis of mitochondria at the ID, longitudinal sections of ID regions were used if the ID and neighbouring sarcomeres were reasonably well ordered and the Z-discs clearly seen. The width of the cell at the ID was noted. A stepped imaginary line was drawn across the image at the level of the first Z-disc from the edge of the ID. The number of columns of mitochondria crossed by this line was counted. The same was done for a line at the edge of the ID (the TrJ) and again for the same positions the other side of the ID. The number of columns of mitochondria that continued across the ID was noted. Between 50 and 250 μm of ID were analysed for each specimen.

## Results

### Organisation of mitochondrial columns and their relationship with t tubules

The organisation of myofibrils and columns of mitochondria running in parallel throughout the cardiomyocyte can be seen in Fig. 1. Within the column the mitochondria are closely apposed with little space between them. The columns are frequently partly or wholly interrupted at the level of the Z-disc by transverse tubules. The figure shows aspects of the t tubule system that have been described previously, including single and double transverse tubules as well as some axial and oblique ones (Fawcett and McNutt 1969; Forssmann and Girardier 1970; Sperelakis and Rubio 1971; Forbes et al. 1984; Tomita and Ferrans 1987; Forbes and van Neil 1988). It is clear that most of the transverse tubules found at the mitochondrial/myofibrillar Z-disc junctions are of large diameter (arrow Fig. 1). The transverse tubules between myofibrils tend to be of a smaller diameter (arrowhead). Oblique tubules run through the mitochondrial columns from one Z-level to another when the fibrils are not in axial register.

**Fig. 2:**
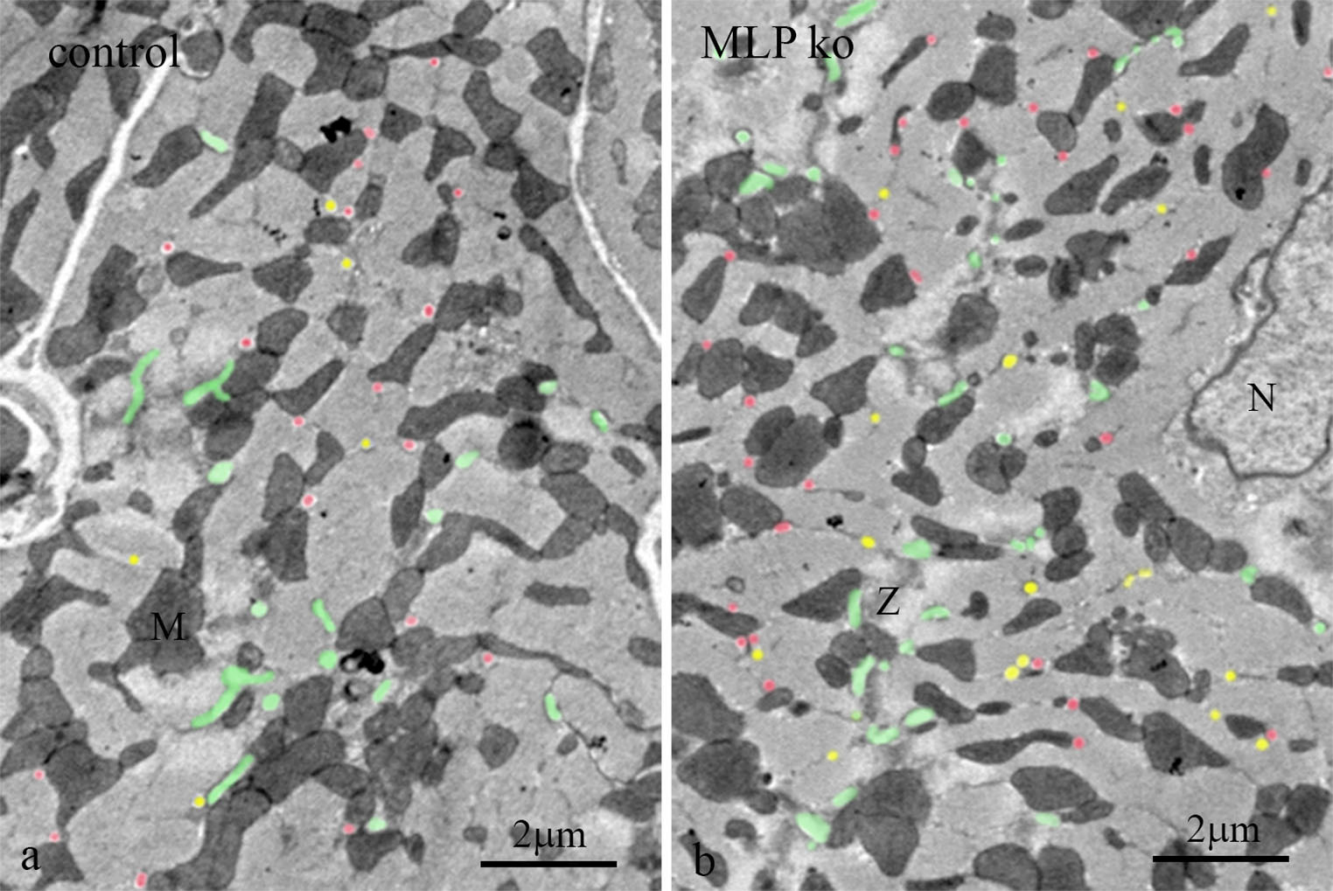
Transverse sections of mouse cardiomyocytes showing the distribution of myofibrils, mitochondria and t tubules in control (**a**) and MLP KO (**b**). *Dark lines* of Z-discs (Z) flanked by lighter I-band regions cross the cell. *Green* elongated shapes show slices through transverse tubules within the I band region. *Red* and *yellow circles* indicate axial tubules in the A-Band. *Red dots* indicate those seen at mitochondrial/myofibrillar borders and *yellow dots* show those between myofibrils. (Color figure online)

Axial tubules run between transverse tubules for at least one sarcomere (Soeller and Cannell 1999; Wagner et al. 2012). Their circular profiles can be identified most easily in transverse sections through A-band regions where tubules of other orientations are absent. (Fig. 2a) (Fawcett and McNutt 1969). We counted them in relation to the area of A-band involved, in a number of cells from 3 month and 9 month papillary muscle (Table 1). We found that the density of axial tubules was ~0.16/μm^2^. One possible error in this measurement was whether all axial tubules were counted. At the magnification used, tubules greater than ~60 nm in diameter were clearly observed. Soeller and Cannell’s recent observations using high resolution optical methods give a range of 20–500 nm for all tubules with very few below 60 nm and an average of 200 nm (Soeller and Cannell 1999). Essentially all of the axial tubules measured here fell in the range of 80 -180 nm (average ~130 nm), significantly below this value but in line with other EM observations (Forssmann and Girardier 1970; Pinali et al. 2013). One possible reason for this difference is that the axial tubules may be smaller in diameter than the transverse tubules (Ayettey and Navaratnam 1978). In Fig. 2a the transverse tubules are seen as elongated (green) profiles in the I-band region. Measurements of their smallest dimension gave an average diameter of 180 nm (range 110 nm -270 nm) in the range found by Soeller and Cannell and similar to the value of 200 nm obtained for transverse tubules by STED microscopy (Wagner et al. 2012).

**Table 1:** Characteristics of axial tubules (AxTs) in control and MLP KO hearts

	3 m con	9 m con	3 m MLP	9 m MLP
Average density of AxTs/um^2^	0.16 SD 0.07^a^ N = 19 cells	0.15 SD 0.05^c^ N = 5 cells	0.28 SD 0.15^b^ N = 27 cells	0.26 SD 0.15^d^ N = 16 cells
Ratio of near mitochondria AxTs to total	0.79 SD 0.14 N = 19	0.71 SD 0.1 N = 5	0.75 SD 0.12 N = 27	0.68 SD 0.18 N = 16
AxT diameter nm	132 SD 36 N = 66	122 SD 38 N = 50	131 SD 38 N = 76	113 SD 28 N = 57

Significance of difference; ^a^ and ^b^ *P* = 0.001; ^c^ and ^d^ *P* = 0.04

One obvious feature of the distribution of the axial tubules was that, while all were at the edge of a myofibril, many were also in close proximity to mitochondria. In Fig. 2 those sandwiched between myofibrils and mitochondria are shown in red, whereas those with myofibril neighbours only are coloured yellow. None were found between mitochondria. The number of red (myofibril/mitochondria) tubules was >70 % of the total (Table 1). To determine how this related to the cellular organisation, we needed to know the number and size of myofibrils and mitochondria. Theakston and colleagues (Theakston et al. 2010) determined these numbers in transverse sections of a few cells in rat heart and showed that, although mitochondria occupy less space (35 %) than myofibrils, they are only about half the size, and therefore there are more of them. Using this data and assuming a random distribution of mitochondria, it can be calculated that the combined circumferences of myofibrils is approximately the same as that of the mitochondria. Considering two neighbouring areas, the chance that they are both mitochondria is 1/2 × 1/2 = ¼, as is the chance that they are both myofibrils. The chance that they are myofibril and mitochondria is 1/2 × 1/2 × 2 = 1/2. As a result, two-thirds of a myofibril is bounded by mitochondria on average. If the axial tubules were randomly distributed around the myofibrils, then some 2/3rds would also have a mitochondrion as a neighbour, a value similar to what we have found.

### Organisation of mitochondria at the intercalated disc

**Fig. 3:**
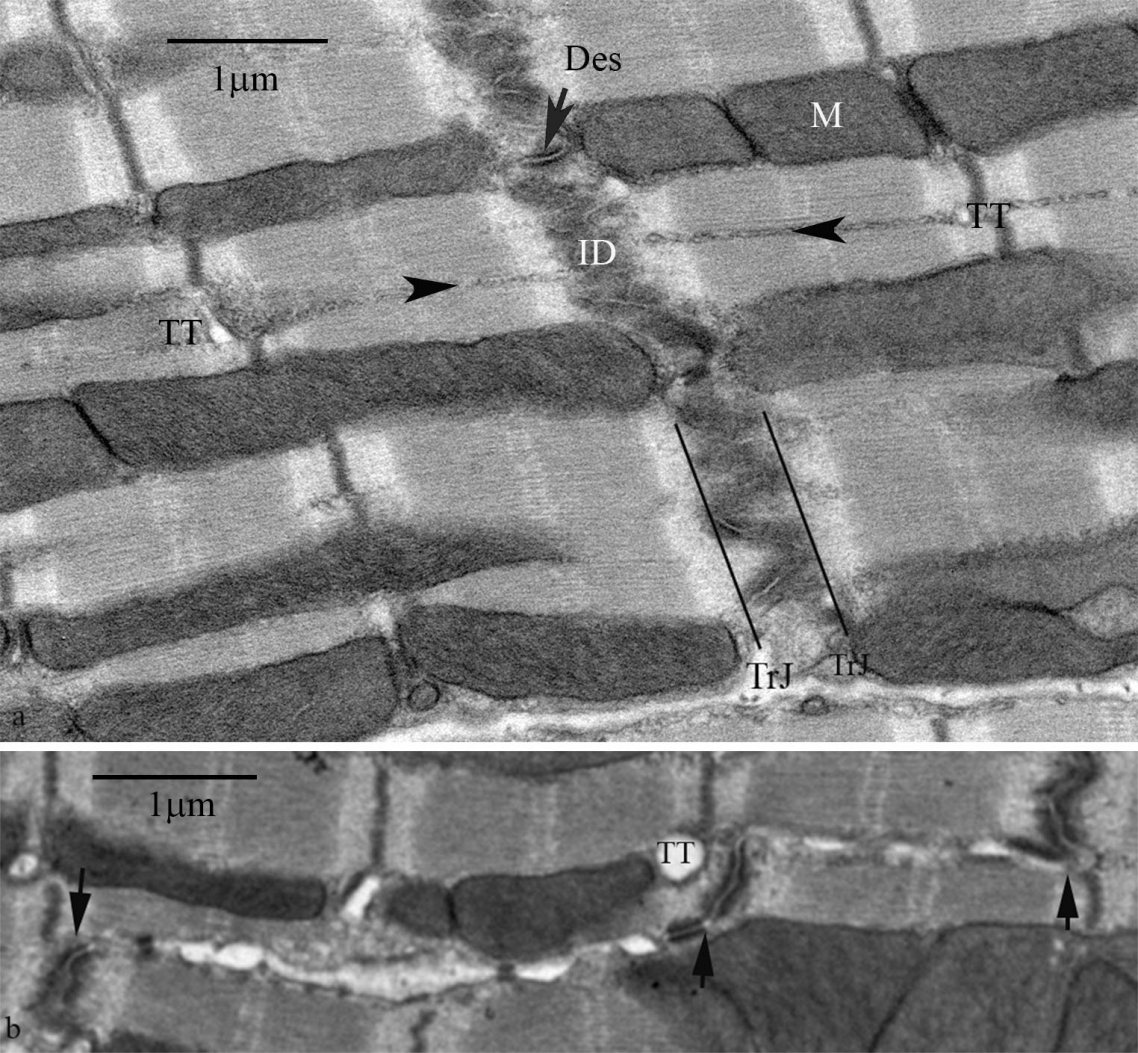
Electron micrographs of longitudinal sections of mouse ventricular myocytes at the level of the ID. **a** Section showing the presence of mitochondria up to the edge of the ID and the continuity of fibrils and columns of mitochondria to either side of the ID. M, mitochondria; TrJ, transitional junction; Des, desmosome; *arrowheads* indicate SR strings; TT transverse tubule. **b** Section to show the presence of transverse tubules at the Z-disc near where a step in the ID occurs, but not at the ID itself. *Arrows* indicate steps in the ID; TT, transverse tubule

**Fig. 4:**
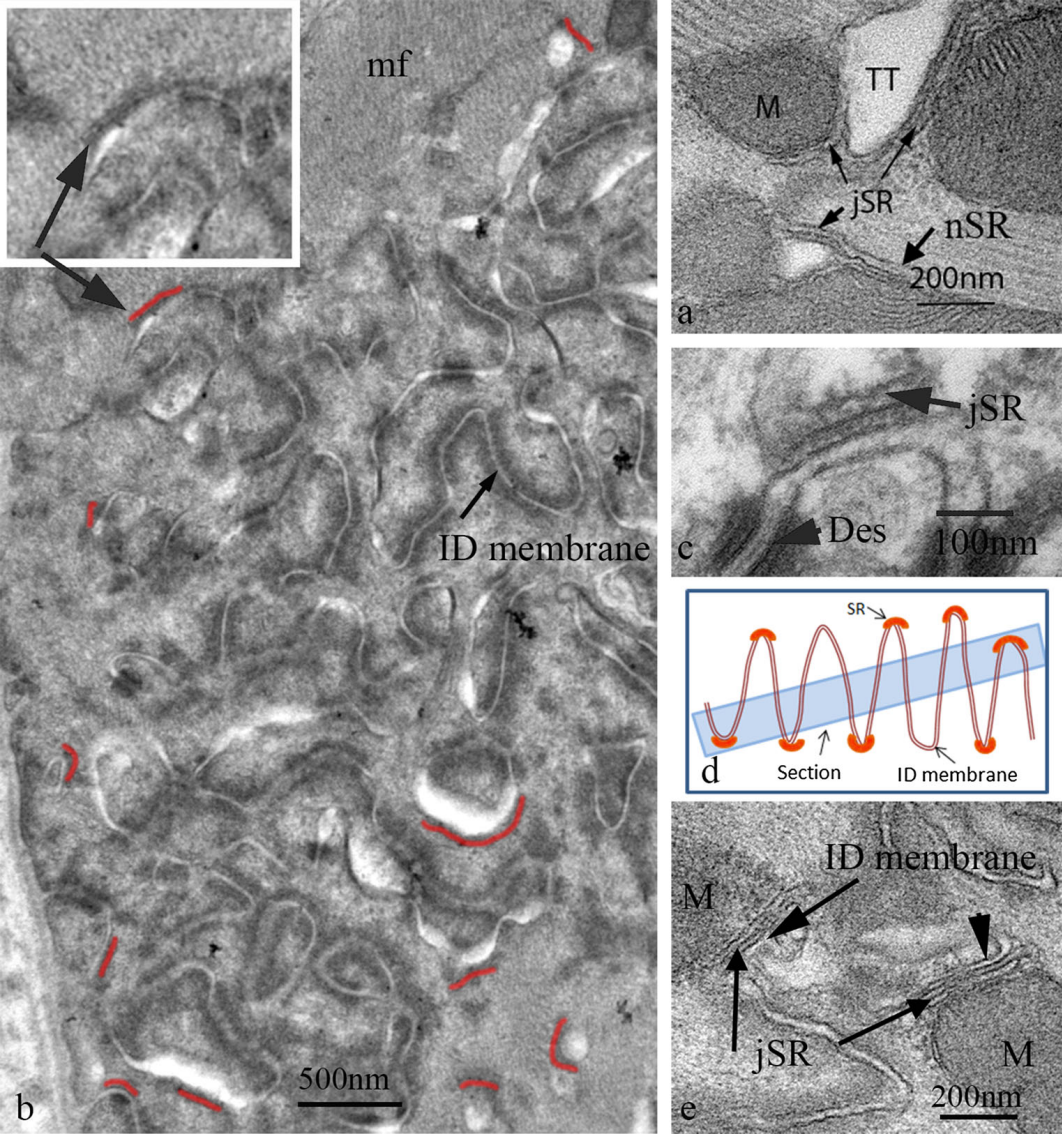
**a** Electron micrograph of mouse papillary muscle showing the organisation of SR, mitochondria (M) and transverse tubules (TT) at a Z-disc. Note the jSR partly sandwiched between the TT and the mitochondria. **b** Slightly oblique transverse section through a large ID in papillary muscle from a 9 m animal showing the circular profiles of cross sections of the ID folds. No mitochondria are found in this area. The jSR vesicles associated with the ID membrane are shown in *red*. They are only found at the periphery of the imaged ID. *Inset* shows indicated area without colour to show the SR vesicle. mf, myofibrils. **c** High magnification image of jSR vesicle complexed with the top of an ID fold. The SR shows the flat shape and protein-rich regions both within the vesicle and between SR and ID membrane characteristic of cardiac peripheral and t-tubule associated jSR. **d** Diagram to show that in a slightly oblique section of an ID the SR at the tops of the ID peaks would only be seen at the edges of the imaged ID region. **e** Electron micrograph showing jSR sandwiched between the ID membrane and mitochondria (M) in longitudinal section. (Color figure online)

At the ends of the cardiomyocyte the plasma membrane is deeply folded, the folds of neighbouring cells interdigitating to form the ID. Figure 3a shows that both myofibrils and mitochondrial columns run up to the ID. The myofibrils maintain their order up to the margin with the ID and the two meet at the transitional junction (TrJ) (Bennett et al. 2006, 2012). The mitochondrial columns also were found to continue to this junction and did not generally penetrate into the ID folds. Their absence in the ID was clearly revealed in transverse sections (Fig. 4a).

Figure 3a also shows the continuity of the organelles between neighbouring cells. Like the myofibrils, the mitochondrial columns tended to continue axially at the same point on the other side of the ID in the neighbouring cell. In general, at least 60 % of the mitochondria columns reaching the ID were matched in the abutting cell. The fibril continuity occurs because the thin filaments from the terminal sarcomeres extend into the ID fold and invest in the adherens (composite) junctions at the ID membrane where the contractile force is transduced from one cell to the next. The ID membranes where mitochondrial columns abut were usually welded together by desmosomes (Fig. 3a). These observations suggested that mitochondrial and myofibril growth was closely coordinated both within a cell and between cells across the ID.

### Relationship of SR/t tubules with mitochondria and the ID

Flattened vesicles of jSR form dyads by wrapping around both transverse (Fig. 4a) and axial tubules (Asghari et al. 2009). The flattened shape of the jSR vesicle and its internal density is characteristic of the heart (Franzini-Armstrong et al. 2005) unlike in the triad of skeletal muscle where the SR vesicle is more dilated. Such vesicles are frequently, at least partly, insinuated between mitochondrion and transverse tubules (Fig. 4a) (Sharma et al. 2000), showing the importance of dyad/mitochondrial proximity. The free SR which surrounds the fibrils is formed of more open, less dense, vesicles, some of which are sandwiched between the fibril and the mitochondria. The SR was not seen to penetrate between mitochondria in a column except when associated with a tubule.

It is of note that transverse tubules were not seen at the TrJ/ID, although they were positioned normally at the first neighbouring Z-disc level. Also, where there was a step in the ID and a Z-disc was in close proximity to the ID edge, a transverse tubule was seen (Fig. 3b). However, free SR vesicles continues to coat fibrils and mitochondria up to the ID (Sommer and Waugh 1976). Here jSR is found associated with ID membrane. As recently described (Leo-Macias et al. 2015) this structure is similar to the peripheral jSR couplons/CRUs frequently found at the lateral plasma membrane and in all obvious respects resembles the flattened vesicles associated with transverse tubules. In particular, they are the same in their width, 25–30 nm, their foot density of RyR between SR and membrane and their internal particulate density attributed to a calsequestrin/junctin/triadin complex (Tijskens et al. 2003) (Fig. 4c). We observed that the jSR vesicles were found at the tops of the membrane folds which were devoid of other structural domains (Fig. 4c–e). They were rare deeper within the ID folds, even those of large amplitude. This relationship was clearest in slightly oblique transverse sections where circular profiles of the tops of the folds were seen at the edges of the ID region. Here vesicles with the characteristic flattened shape and membrane apposition of jSR were seen (coloured red in Fig. 4b, d). Some of these SR vesicles were sandwiched between the ID membrane and mitochondria (Fig. 4e).

### Electron tomography of SR at the ID: relationship with mitochondria

**Fig. 5:**
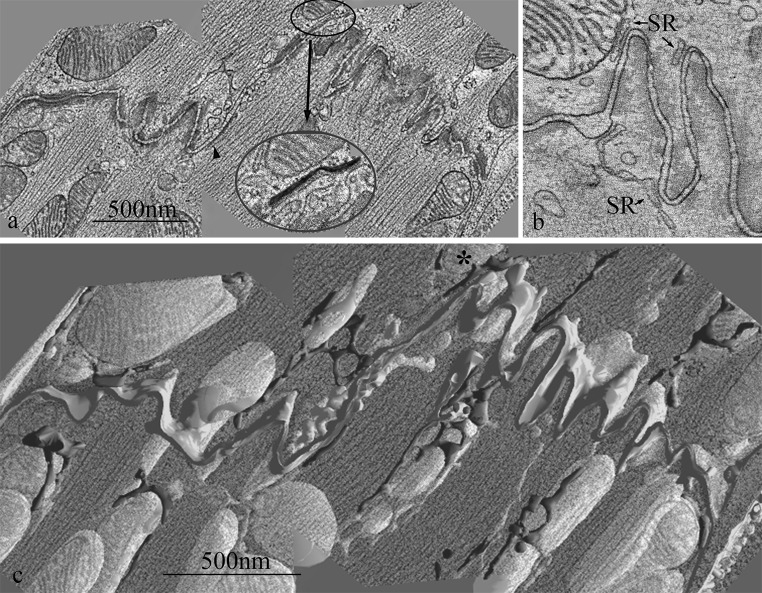
Electron tomograms of mouse papillary IDs. **a** One plane through a tomogram obtained by joining 3 tomograms side by side showing a stepped ID. Two jSR vesicles are highlighted. One is seen applied to ID membrane on the step (*arrowhead*) and another between ID membrane and mitochondrion (*circled*). *Inset* Area in circle in (**a**) enlarged to show detail. jSR coloured *red*. **b** Plane through another tomogram showing SR vesicles associated with ID membrane and mitochondrion and stretching between two peaks at the ID. **c** Segmented model showing whole volume of tomogram in (**a**) superimposed on one central plane of the original. Mitochondria (*yellow*), SR (*red*) and ID membrane (*green*). Note that the image and the mitochondria are partly transparent so that SR red becomes pink below the image and gold behind mitochondria. *Asterisks* indicates mitochondrion in *inset* in (**a**). (Color figure online)

To understand the relationship between mitochondria, SR and ID membrane more clearly, we carried out electron tomography. Figure 5a shows one level of a tomogram of an extended region of ID composed of two lengths of plicate membrane and a step of one sarcomere between them. The complex pleating of the ID membrane is clearly illustrated in the tomogram (see also Leo-Macias et al. 2015; Pinali et al. 2015). SR, when present, is principally associated with areas’bare’ of structural domains and in one place can be seen between a mitochondrion and the ID membrane forming an ID-jSR-mitochondrion complex (Fig. 5a, inset). A similar area from another tomogram again shows a close juxtaposition of mitochondria, SR and ID membrane (Fig. 5b). It also shows how the jSR/ID membrane nodes are connected between ID peaks via the more dilated strand of free SR. The spacing between the outer mitochondria membrane and the SR is seen to be similar to the dyad spacing, consistent with linkers of 10–15 nm (Boncompagni et al. 2009).

A 3D model formed from the segmented image stack of Fig. 5a is seen in Fig. 5c. Mitochondria (yellow), SR (red) and ID membrane (green) are shown but myofibrils have been omitted for clarity. SR forms a branched network around both fibrils and the mitochondrial columns. The network extends towards the ID membrane and associates with the tops of the folds. In places it reaches over the top of mitochondria and inserts itself between the mitochondria and the ID membrane as seen in Fig. 5a inset. We rarely observed direct contact of mitochondria with ID membrane. It is the SR which is the intermediary between the mitochondrial columns and the ID membrane.

### Mitochondria, t tubules and SR in MLP KO hearts

**Fig. 6:**
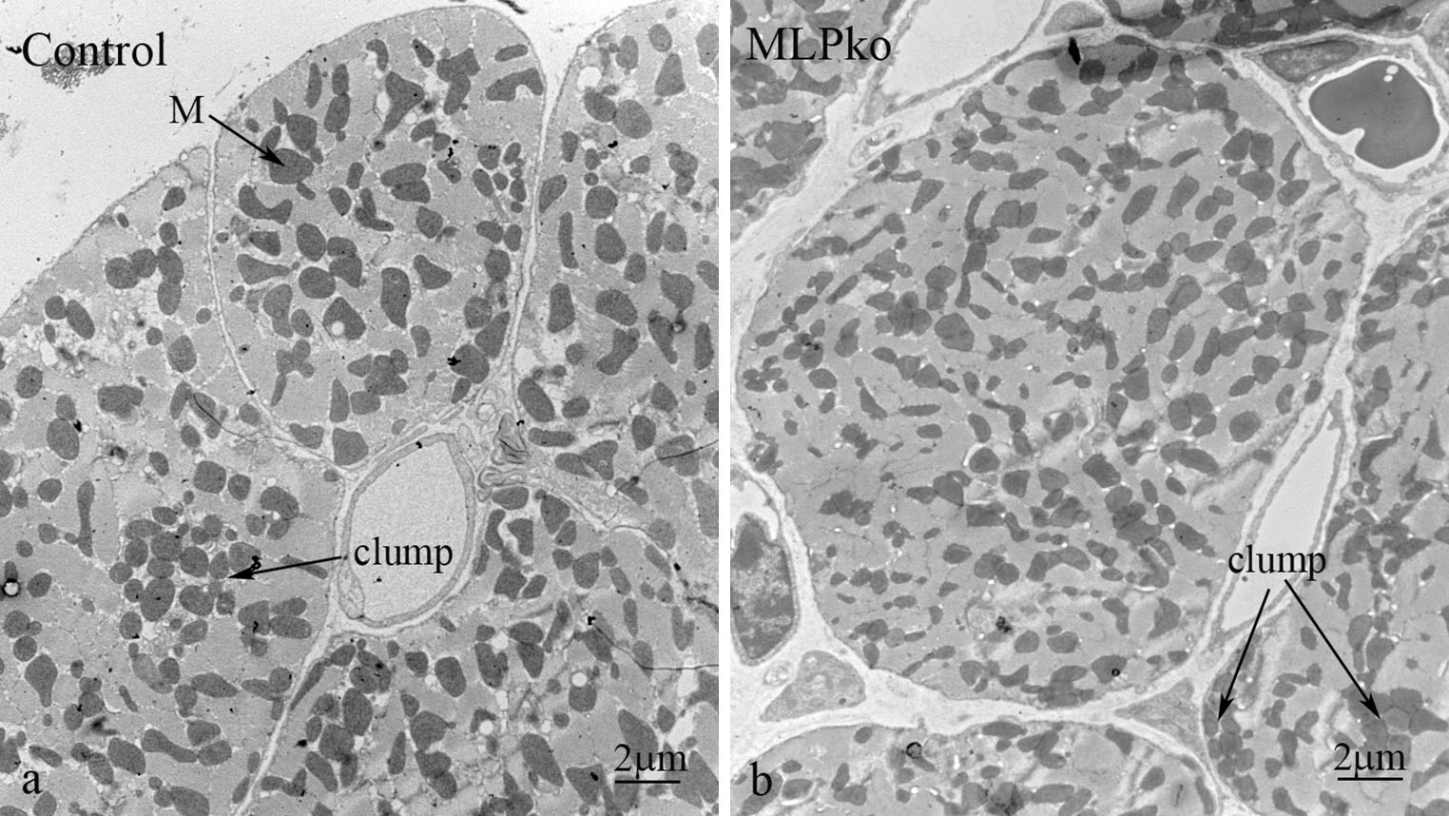
Transverse sections of 3 month old papillary muscles showing distribution of mitochondria (M). **a** Control. **b** MLP KO. Clumps (*large arrows*) of mitochondria are seen in both. There appears to be a bigger range of mitochondrial sizes in the null sample with a larger number of small ones (*small arrows*)

The MLP KO mouse model for dilated cardiomyopathy has a mild phenotype which appears to stabilise after maturity is reached and the animals attain normal old age. At 10 weeks the heart is ~50 % heavier than the control and the end stage diastolic diameter 20 % greater, with an accompanying loss of function (Arber et al. 1997). It has been noted that there are changes in mitochondrial organisation and function. Clumping of the mitochondria has been observed in sub-sarcolemmal groups, as has disorder in the columns and the presence of smaller mitochondria (van den Bosch et al. 2005; Wilding et al. 2006). On an observational basis there is disagreement about the numbers of mitochondria present; one group suggesting a loss (van den Bosch et al. 2005) and another suggesting no change (Wilding et al. 2006). Although there are regions of structural disorder and fibrosis in these hearts, most of the cells are reasonably well ordered. So much so, that the density of mitochondria we saw in transverse sections through the body of control and MLP KO cardiomyocytes did not seem obviously different (Figs. 2, 6) (see below for exceptions). There did, however, seem to be a bigger range of mitochondria sizes in the MLP KO (Fig. 6). Since the mitochondria were much more electron dense an estimate of their concentration was obtained by determining the percentage of darker pixels in the cell. The results from papillary muscle of 4 age-matched pairs of animals from 3 m to 9 m showed that on average ~35 % of the area of the cell was occupied by mitochondria both in control and MLP KO papillary muscle, and that this was independent of age (Table 2). From this we concluded that there is no significant difference in the volume of mitochondria in the MLP KO compared to wild type in agreement with Wilding et al. (2006).

**Table 2:** Area of mitochondria (%) in transverse sections of control and MLP KO papillary muscles

Age	3 m	6 m	9 m
Control	35 % SD 3.5 % N_A_ = 2, N_C_ = 29	35 % SD 2.3 % N_A_ = 1,N_C_ = 9	35 % SD 3.4 % N_A_ = 1, N_C_ = 10
MLP KO	34 % SD 3.3 % N_A_ = 2, N_C_ = 47	37 % SD 4.7 % N_A_ = 1, N_C_ = 28	33 % SD 6.1 % N_A_ = 1, N_C_ = 27

*N*_*A*_ Number of animals; *N*_*C*_ Number of cells

**Fig. 7:**
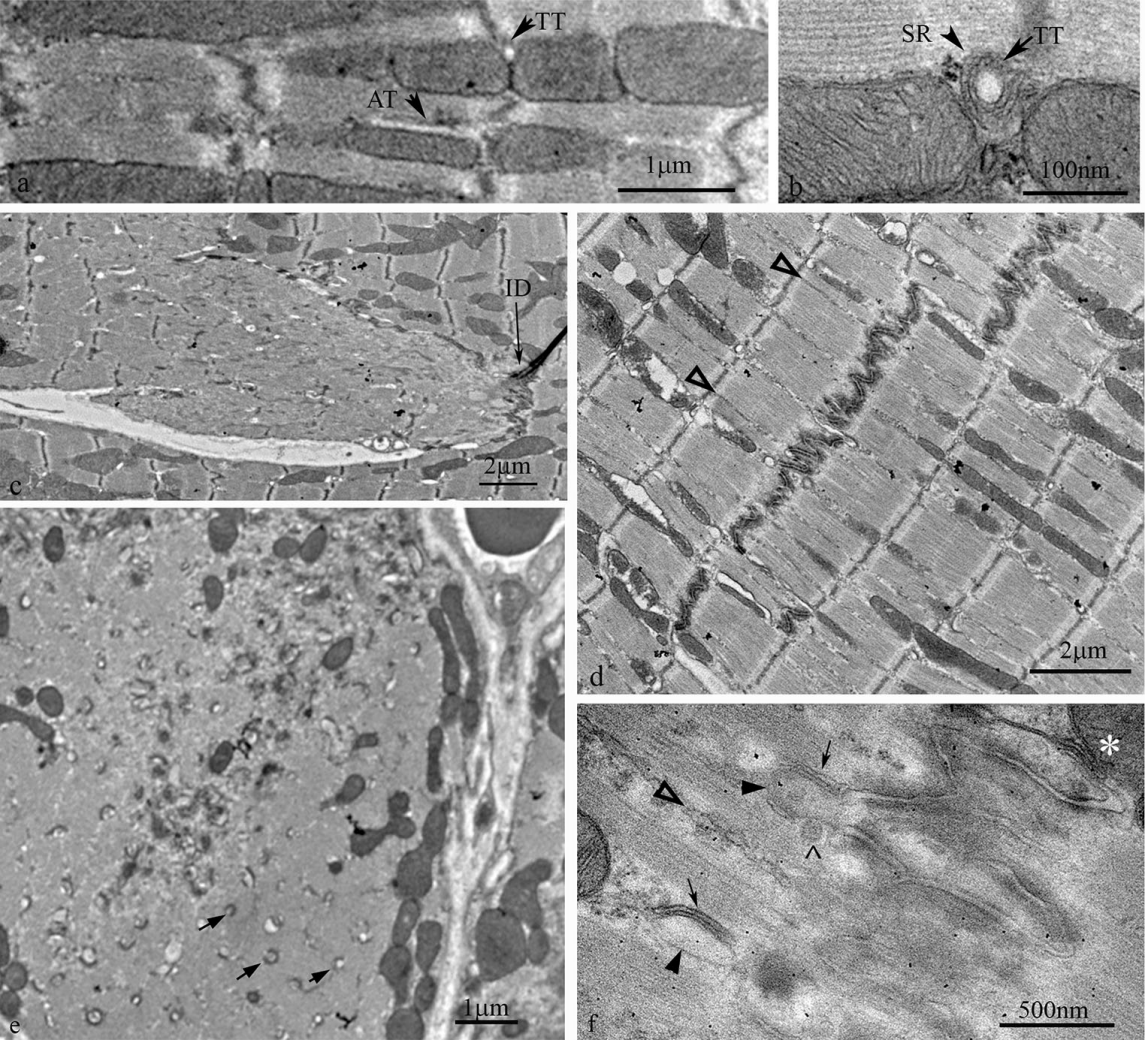
Electron micrographs of MLP KO *left* ventricle. **a** Longitudinal section of part of a cardiomyocyte showing transverse (TT) and axial tubules (AT). **b** jSR and t tubule sandwiched between mitochondria. **c**–**f** areas near IDs showing changes in the distribution of mitochondria compared to control muscle. **c** Disordered sarcomeres and absence of mitochondria in a region of one cell near the ID. **d** Cell with good order but an absence of mitochondria within one sarcomere to either side of the ID. *Open arrows* indicate rows of SR vesicles between fibrils up to the ID. **e**. Low magnification view of a transverse section from 9 m LV. The cell on the *left* shows an ID and a neighbouring mitochondria-sparse region containing a number of membrane fingers of axial tubules (*arrows*). The few mitochondria present are small compared to those in the neighbouring cell. **f** Higher magnification view of part of an ID showing extensions of ID folds into the A-band region of the proximal sarcomere (*arrowheads*). They are coated with jSR (*arrows*). *Open arrow* indicates a string of free SR; *accent symbol*, coated vesicle; *asterisks*, mitochondrion/jSR/ID membrane complex

It has been reported that there is a loss and/or increase in disorder of transverse tubules in DCM, hypertrophy and heart failure (Ong and Hausenloy 2010; Ibrahim et al. 2011). While we cannot be quantitative about the number of tubules present, we did not detect an obvious change in distribution or loss of transverse tubules at the Z-disc level in the MLP KO heart (Figs. 2b, 7a). This may be because of the mild phenotype. There was, however, more axial disorder in the disposition of the myofibrils, which could lead to more oblique tubules. A better estimate of the number of axial tubules could be determined in transverse sections (Fig. 2b; Table 1). Monitoring the axial tubules seen in the A-band only, the average diameter was essentially the same as in controls (average 130 nm; range 70–190 nm). The major difference between the control and the KO in age-matched pairs was that the density of axial tubules in transverse sections was more than 50 % greater than in control hearts (~0.25/μm^2^ compared to 0.16/μm^2^). However, the relative proportion associated with mitochondria/myofibrillar interfaces, was ~2/3, compared to myofibril alone, 1/3, essentially the same as that seen in control cells.

No difference in the disposition of SR in its relationship to mitochondria, t tubules or myofibrils was observed in the MLP KO heart compared to control in the bulk of the cell; jSR was frequently found sandwiched between mitochondria and t-tubules at the Z-disc level (Fig. 7b), and free SR was seen at the edge of fibrils (Fig. 7d).

### Mitochondria at the ID in MLP KO heart

It has been reported that in the MLP KO heart there is a loss of mitochondria close to the ID even in apparently normal looking cells (Wilding et al. 2006; Wilson et al. 2014) (Fig. 7c−f). This loss may be related to structural changes at the ID in DCM which have been previously described (Arber et al. 1997; Ehler et al. 2001; Perriard et al. 2003; Wilson et al. 2014; Pluess et al. 2015). In the MLP KO these changes include an increase in the amplitude of the ID folds and the inclusion of extra sarcomeres across the cell. In extreme cases, profligate growth of disordered sarcomeres together with the absence of mitochondria was seen at the ends of the cell, while the rest of the cell and its neighbours appeared normal (Fig. 7c) (Wilson et al. 2014). In cells where the sarcomeric structure was clearly seen the absence of mitochondria appeared to be confined to a region of about one sarcomere to either side of the ID (Fig. 7d). To quantify this, counts of the number of mitochondria columns at different axial positions in relation to the ID were obtained from 5 samples of LV and papillary from two age-matched pairs of normal and KO mice (see “Measurements” section). The age-pooled data showed that in both sample types the number of columns of mitochondria at the first Z-disk from the ID was on average ~0.5/μm (control 0.53 ± 0.12; MLP KO 0.54 ± 0.04, *P* = 0.9). Of those a higher proportion (~80 %) continued to the TrJ at the edge of the ID in control animals compared to the KO (~60 %) (control 0.44 ± 0.08; MLP KO 0.31 ± 0.04 *P* = 0.03).

This suggested that the normal strong coordination of mitochondria and myofibril growth at the ID is compromised in the knock-out hearts. Furthermore, significantly fewer of the columns in the KO had counterparts on the other side of the ID, as little as 40 % of those reaching the TrJ compared to 60 % in the controls (control 0.28 ± 0.07; MLP KO 0.12 ± 0.06; *P* = 0.005). Thus, the continuity of the mitochondrial columns across the ID was lost, further evidence that communication between cells in the KO was affected.

Van den Bosch et al. noted that the mitochondria in the MLP KO cardiomyocytes were ~30 % smaller than in the control animals (van den Bosch et al. 2005). A considerable variation in size can be seen in Fig. 6, but the size difference is particularly obvious near the ID (Fig. 7e).

### SR at the ID in MLP KO mouse

Absence of mitochondria at the ID may lead to changes in SR disposition. As in control cells there were longitudinal strings of free SR reaching along and around the sides of the myofibrils towards the peaks of ID membrane (arrows Fig. 7d, f). At the ID the relationship with the sparse mitochondria was apparently normal. The asterisk in Fig. 7f shows jSR sandwiched between ID membrane and mitochondria. One structural feature that was not observed in control hearts was that the long folds of the ID sometimes protruded beyond the TrJ limits of the ID and penetrated into the A-Band region of the proximal sarcomere (Fig. 7f, arrows). In some cases they opened out into bulbous loops of membrane bounded by jSR. In other cases fingers of ID membrane seemed to stretch into the A-band, again bounded by SR. In the transverse section in Fig. 7e, outside of the very convoluted ID membrane region, and in a region lacking mitochondria there are many round profiles that resemble axial tubules seen in the body of the cell. These may be remnant features of sarcomere growth at the ID when mitochondria are not produced.

## Discussion

### T tubules and mitochondria

High resolution optical methods have been used recently to obtain a three dimensional view of the t tubule system (Soeller and Cannell 1999; Wagner et al. 2012). Transverse, oblique and axial orientations of tubules have been quantified in mouse and rat ventricle. Although seen in the substantial literature of earlier electron microscopy observations, the recent work has identified a surprising number of oblique and axial tubules which make connections from one axial level of transverse tubules to the next. The optical data does not take into account the unseen mitochondria and their relationship to this organisation. Our observations, like others, suggest that most of the transverse tubules are related to the Z-disc/I-band and very frequently are localised close to mitochondria (Ramesh et al. 1998). Although it appears so, the fibrils in the cardiomyocyte are not always axially in register across its whole width but sometimes have a helicoidal arrangement (Jayasinghe et al. 2010). At the dislocations caused by this arrangement, the fibrils are axially displaced and the t tubule needs to adopt an oblique path in order to connect the Z-discs of neighbouring fibrils. We have found that the path of these tubules is usually through a column of mitochondria. This arrangement is illustrated clearly in the guinea pig heart (Sperelakis and Rubio 1971). Cutting through the mitochondrial columns in this way makes the necessary axial shifts more gradual.

Axial tubules play a role in connecting transverse tubules at different axial levels. They have a smaller diameter than most of the transverse tubules, as has been pointed out by Ayettey and Navaratnam who described 4 subsets of tubules of different diameters in the rat myocardium (Ayettey and Navaratnam 1978). Soeller and Cannell estimate that 40 % of the length of tubules in the rat cardiomyocyte is outside of the I band region and is oblique/axial (Soeller and Cannell 1999), while Wagner et al. report that about a third of the tubules in mouse adult cardiomyocytes are axial (Wagner et al. 2012). It is not easy to relate our estimates of axial tubule density to these data. We found that there was ~one axial tubule/6 μm^2^ in transverse sections. Since we know that the major transverse tubule component comes from the sarcolemma on an approximately square lattice 2 μm axially × 2 μm azimuthally (Soeller and Cannell 1999), it would need one axial tubule to branch off a transverse tubule every 3 or 4 μm. This value does not seem unreasonable when viewing the light microscope images.

All axial tubules we observed had a myofibril neighbour, at least 2/3 of which were close to mitochondria. These data are consistent with the idea that the axial tubules can occur with equal probability anywhere at the edge of myofibrils. The fact that the myofibrils abut mitochondria for much of their circumference, means that a high proportion of axial tubules also have a mitochondrial partner.

Wagner and colleagues have shown that, in a model for heart failure by myocardial infarction (MI), the length of all classes of tubules increased (Wagner et al. 2012). In particular, axial tubules increased by 100 % after a 4 weeks. This is not dissimilar to our observations on the MLP KO mouse where we found a 60 % increase in the number of axial tubules. In general, an increase in t tubules is unexpected since the organisation of tubules in the cardiomyocyte has been shown to be severely disrupted in many examples of heart disease (see e.g. Ibrahim et al. 2011). This is often identified by a reduction in the strength of the transverse tubule axial repeat seen by a plasma membrane dye and a loss of the tubule outlets at the plasma membrane. The MLP KO mouse, although exhibiting a DCM phenotype, is long-lived and clearly is not suffering from major detubulation. However, the axial disorder of the fibrils which would lead to an increase of the obliquity of the tubules reaching from one fibril to the next and the increased number of axial tubules would reduce the relative power of the axial repeat. These are likely to be general contributing factors to observed tubule disorganisation in heart disease.

### Importance of SR in heart

The complex meshwork of SR writhing through the cardiomyocyte has been described in 3D by scanning EM (Pinali et al. 2013). It is found close to and interacts with three main structures in the cell, the myofibrils, the plasma membrane/t tubules and the mitochondria. The connection to the myofibril involves at least an actin-based mechanism at the Z-disc (Kee et al. 2009) and an obscurin/ankyrin B complex at the M-line (Cunha and Mohler 2008; Lange et al. 2009). The most well characterised SR interaction is that of jSR vesicles forming couplons/CRUs on the peripheral cell membrane and t tubules. These CRUs also occur at the ID, where we have found them to be localised to the tops of the ID membrane folds, thus ensuring the continuity of the SR from one end of the cell to the other. The extracellular environment of the intercellular ID cleft has, like the t-tubule, a high calcium concentration. This is necessary to maintain the strength of bond between cadherins of the adherens junctions and desmosomes. In the absence of t tubules the presence of CRUs here would supply the need for calcium influx into this region of the cell: for contractile activity of the proximal half sarcomere, for mitochondria function and for the many, mostly unknown, functions of the components in the ID folds themselves.

The SR relationship with mitochondria is less well characterised. Our tomography has shown how the network of SR appears to encapsulate the mitochondrial columns even near the ID. This association is in agreement with the observation of physical tethers between mitochondria and both free and jSR (Boncompagni et al. 2009; Hayashi et al. 2009) and is supported by the observation that in fibroblasts a subset of ER is bound to mitochondria (de Brito and Scorrano 2008). As we have seen, the high density of mitochondria in the heart and the random distribution of fibrils and columns lead to a large proportion of the myofibril surface abutting a mitochondria neighbour. This allows mitochondria access to some two-thirds of the SR network.

The SR connection to both mitochondria and cell membrane comes together most obviously where mitochondria come close to CRUs. 90 % of CRUs have been found to be within a few hundred nm of mitochondria (Ramesh et al. 1998; Sharma et al. 2000; Boncompagni et al. 2009). This proximity allows mitochondria local periodic access to high levels of calcium during the excitation/contraction cycle. Often the association is much closer, in particular, where the flattened jSR sacs are, at least partly, sandwiched between mitochondria and t tubules, peripheral or ID membrane. In other cell types it is suggested that there are direct routes for calcium access at the mitochondria/ER interface. In particular, Csordas and colleagues have shown that the length of tether between outer mitochondrial membrane (OMM) and the ER is important for calcium loading of the mitochondria (Csordas et al. 2010; Marchi et al. 2014). Possibly this could occur in the heart although it is not clear what the composition of the observed tethers is or, indeed, whether the same protein is present in the links to free and jSR. One possibility is mitofusin 2 which has been located between mitochondria and ER (SR) and associated with inter-organelle calcium signalling domains (de Brito and Scorrano 2008; Chen et al. 2012; Song and Dorn 2015).

Whatever the composition of the links seen in the heart, they certainly appear to contribute to the structural stability of the t tubule/SR/mitochondria complex and may play a role in SR fission of mitochondria. Even in the tightly packed cardiomyocyte, mitochondrial fission and fusion can occur, albeit slowly, over several hours (Huang et al. 2013). The fission process involves ER which attaches to a mitochondrion through a DRP-1 containing complex (da Silva et al. 2014; Marchi et al. 2014). Where the ER wraps around it the mitochondrion is fragmented in an actin-dependent manner. It is possible that this process is involved in the heart to produce breaks for t tubule insertion in the mitochondrial columns particularly at the myofibril Z-disc level.

### The MLP KO and mitochondria function

There is considerable evidence for changes in mitochondrial function in heart disease (Ong and Hausenloy 2010; Dorn 2015). In our model for DCM, the MLP KO mouse, the organisation of mitochondria is compromised. While we have found no loss of mitochondria volume in the body of the cell, there are some changes in distribution, shape and size (van den Bosch et al. 2005; Wilding et al. 2006). Wilding and colleagues suggest that this cytoarchitectural disorganisation reduces mitochondrial/SR crosstalk and results in a 30 % impairment in direct nucleotide channelling between mitochondria and SERCA (Wilding et al. 2006). The role of MLP is not well understood, although it has been suggested that it is present at the Z-disc and involved in strain detection (Hoshijima 2006). Whatever its role, it is probable that, as in most heart disease, the MLP KO would experience some reorganisation of the t tubules (Ferrantini et al. 2013). Indeed, we found that there was an increase in the number of axial tubules. The changes in tubule organisation and myofibril axial disorder point to a loss of tight control and compact organisation of the structural elements normally present at the Z-level, a result consistent with the proposed role of MLP. This structural relaxation could indeed become apparent as a separation of mitochondria from the myofibril/SR complex, limit the formation of tethers and compromise the energetic crosstalk.

### Mitochondria and cardiomyocyte growth at the ID

The clear organisation of mitochondria columns and myofibrils at the edge of the ID points to their coordinated development both within the cell and from cell to cell during cell growth. This coordination appears to be lost in DCM where structural changes at the ID are most obvious (Perriard et al. 2003). These changes are often accompanied by mutations or changes in expression of ID proteins (Estigoy et al. 2009; Bennett 2015). This is the case for the MLP KO mouse (Ehler et al. 2001). There is increased expression of several adherens junction proteins such as β-catenin and N-cadherin, which is reflected in the increased amplitude of the ID folds and a loss of connexin and gap junctions. Further, as we have seen here, there is a reduction in the number of columns of mitochondria at the level of the proximal sarcomeres close to the ID and a loss of continuity across the ID (Wilding et al. 2006; Wilson et al. 2014). The absence of mitochondria leaves a gap between fibrils that can become filled with extra contractile material. This creates stresses within the cell and across the ID that are not linear and thus leads to the dispersion of tension at the end of the cell and could account for the erratic brush-like growth seen there (Perriard et al. 2003). The loss of coordination from one cell to the other is further reflected in the increased dispersion of size of the MLP KO cardiomyocytes (Leu et al. 2001).

**Fig. 8:**
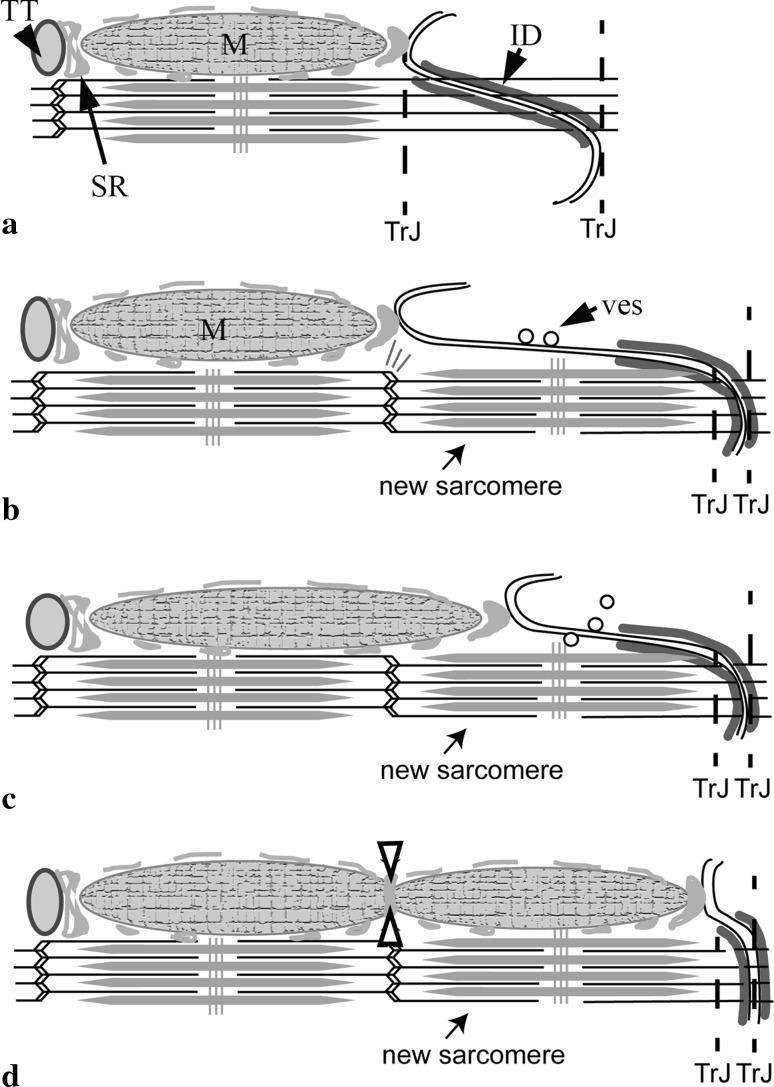
Model of mitochondrial elongation accompanying sarcomere addition at the ID in normal cardiomyocyte growth. **a** to **b** ID folds enlarge to 2 μm and a new sarcomere is inserted (Wilson et al. 2014). *M* mitochondrion; *TT* t-tubule. **b**, **c** The long finger of membrane is removed by endocytosis (ves). The associated SR extends to compensate, allowing room for the mitochondrion to grow. **c** to **d** The ID fold becomes small again and is confined to new TrJ boundaries. Fission of mitochondrion is brought about by SR at the new Z-disc (*open arrows*). (Color figure online)

It has previously been described how sarcomeres could be added to the end of cells within long folds of the ID membrane (Yoshida et al. 2010; Wilson et al. 2014). Our present observations on mitochondria imply that this process should also include them. Comparison of the number of mitochondria columns at the TrJ level with that at the first proximal Z-disc level always shows fewer mitochondria at the TrJ. In the MLP KO this is much more obvious. This would suggest that the development of the mitochondrial columns lags behind that of sarcomere addition. Further observational evidence for this can be seen in the work of Yoshida et al. who show several images of sarcomere incorporation during rapid hypertrophic growth after volume overload. Few if any mitochondria are seen within the growing ID regions. Figure 8 shows how mitochondrial growth might be incorporated into the model we previously proposed (Wilson et al. 2014). There we described a model in which the cardiomyocyte at first grows by increasing the length of the ID folds to 2 μm (Fig. 8a). At this point the elements of a new sarcomere are assembled in the fold with the new Z-disc at the original TrJ (Fig. 8b). After the new sarcomere is incorporated, a long finger of excess ID membrane has to be removed (Fig. 8b). There is evidence that this occurs through endocytosis by coated vesicles (Fig. 7f). The finger of ID membrane retracts to become a new short fold. We propose that the top of the fold remains attached to the SR (via jSR), which grows as the fold shortens, forming attachments to the new sarcomere (Fig. 8c). If this fold was originally associated with a mitochondrial column, this would allow an extension of the old mitochondrion to grow into place and, subsequently, the possibility of it being bisected by the SR at the new Z-disc (Fig. 8d).

This model is supported by the situation at the MLP KO ID. Here the SR continues to the ID folds. However, the absence of the mitochondria sometimes appears to coincide with long folds or fingers of membrane coated with SR that extend into the A-band of the proximal sarcomere. Some of these fingers have bulbous ends which have been shown to contain αII spectrin, a protein found in t-tubules, sarcolemma and at the tops of the folds in the ID (Bennett et al. 2006; Wilson et al. 2014). It seems possible that these are remnant features of sarcomere addition that only slowly retract in the KO cell.

## Conclusions

The role of SR as an essential integrator of cardiomyocyte structure is suggested. It appears to bond myofibril, mitochondria and t tubule in an intimate relationship. Faults that inhibit the growth of mitochondria at the ID lead to morphological changes which affect the ordered association of one cardiomyocyte with its neighbour and lead to heart disease.

## Abbreviations

DCM: Dilated cardiomyopathy
HCM: Hypertrophic cardiomyopathy
MLP: Muscle LIM protein
ID: Intercalated disc
ER: Endoplasmic reticulum
SR: Sarcoplasmic reticulum
jSR: Junctional SR
TrJ: Transitional junction
CRU: Calcium release units
CaV_1.2_: L-type Ca^++^ channel
RyR2: Ryanodine receptor type 2
SERCA: SR calcium channel
